# AMG 900, a potent inhibitor of aurora kinases causes pharmacodynamic changes in p-Histone H3 immunoreactivity in human tumor xenografts and proliferating mouse tissues

**DOI:** 10.1186/s12967-014-0307-x

**Published:** 2014-11-04

**Authors:** Gloria Juan, Tammy L Bush, Connie Ma, Raffi Manoukian, Grace Chung, Jennifer M Hawkins, Stephen Zoog, Richard Kendall, Robert Radinsky, Robert Loberg, Greg Friberg, Marc Payton

**Affiliations:** Departments of Oncology Biomarkers and Early Development, Thousand Oaks, CA 91320 USA; Departments of Oncology Research, Thousand Oaks, CA 91320 USA; Department of Pathology, Amgen Inc, One Amgen Center Drive, Thousand Oaks, CA 91320 USA

**Keywords:** Histone H3, Mitosis, Pharmacodynamics, Cytometry, Tumor biopsies

## Abstract

**Background:**

The Aurora family of serine-threonine kinases are essential regulators of cell division in mammalian cells. Aurora-A and –B expression and kinase activity is elevated in a variety of human cancers and is associated with high proliferation rates and poor prognosis. AMG 900 is a highly potent and selective pan-aurora kinase inhibitor that has entered clinical evaluation in adult patients with advanced cancers. In mice, oral administration of AMG 900 blocks the phosphorylation of histone H3 on serine-10 (p-Histone H3), a proximal substrate of aurora-B and inhibits the growth of multiple human tumor xenografts, including multidrug-resistant models.

**Methods:**

In order to establish a preclinical pharmacokinetic-pharmacodynamic (PK-PD) relationship for AMG 900 that could be translated to the clinic, we used flow cytometry and laser scanning cytometry detection platforms to assess the effects on p-Histone H3 inhibition in terms of sensitivity, precision, and specificity, in human tumor xenografts in conjunction with mouse skin and bone marrow tissues. Mice with established COLO 205 tumors were administered AMG 900 at 3.75, 7.5, and 15 mg/kg and assessed after 3 hours.

**Results:**

Significant suppression of p-Histone H3 in mouse skin was only observed at 15 mg/kg (p <0.0001), whereas in mouse bone marrow and in tumor a dose-dependent inhibition was achieved at all three doses (p ≤0.00015). These studies demonstrate that AMG 900 inhibits p-Histone H3 in tumors and surrogate tissues (although tissues such as skin may be less sensitive for assessing PD effects). To further extend our work, we evaluated the feasibility of measuring p-Histone H3 using fine-needle aspirate (FNA) tumor xenograft biopsies. Treatment with AMG 900 significantly inhibited p-Histone H3 (>99% inhibition, p <0.0001) in COLO 205 tumors*.* Lastly, we illustrate this LSC-based approach can detect p-Histone H3 positive cells using mock FNAs from primary human breast tumor tissues.

**Conclusion:**

Phosphorylation of histone H3 is a useful biomarker to determine the pharmacodynamics (PD) activity of AMG 900. FNA biopsies may be a viable approach for assessing AMG 900 PD effects in the clinic.

**Electronic supplementary material:**

The online version of this article (doi:10.1186/s12967-014-0307-x) contains supplementary material, which is available to authorized users.

## Background

The Aurora family of serine/threonine kinases is represented by three paralogous genes: Aurora-A, −B, and -C. Despite a high degree of kinase domain similarity, the subcellular localizations and functions of the auroras are primarily non-overlapping. Aurora-A and -B play critical roles during mitosis in chromosome segregation and cytokinesis, while aurora -C function appears to be restricted mainly to spermatogenesis. Aurora-A is reported to function as an oncogene capable of transforming rodent fibroblast cells in culture. The aurora-A gene (*AURKA*) locus 20q13 is amplified in a subset of cancers including: bladder, breast, colorectal, gastric, liver, ovarian, and pancreatic. In addition, aurora-A and -B expression is elevated in a variety of human cancers, where their overexpression is associated with advanced clinical staging and poor prognosis [1-3].

Treatment of tumor cells with a small molecule inhibitor targeting both aurora-A and -B causes premature mitotic exit without cell division [4]. The resulting undivided 4 N DNA-containing tumor cells can progress through further rounds of DNA replication without division, a process that ultimately induces cell death. This mechanism of silencing the mitotic checkpoint through inhibition of aurora-B (or dual inhibition of aurora-A and –B) is distinct from that of traditional anti-mitotic agents in that cells do not pause or arrest in mitosis [4-7].

AMG 900, a novel ATP-competitive inhibitor of aurora A/B/C, is active in a panel of human cancer cell lines, including: colon, lung, prostate, breast, ovarian, uterine, melanoma, kidney, liver, and hematologic with EC_50_ values ranging from 0.7 to 5.3 nmol/L [8]. Phosphorylation of histone H3 at serine-10 residue (p-Histone H3) is highly correlated with the G_2_ to mitotis (M) cell cycle transition and essential for chromatin condensation [9]. Given that histone H3 is a direct target of aurora-B, measuring serine-10 phosphorylation represents a measurement of enzymatic activity [10]. Robust methodologies have been developed for immunofluorescence based detection of p-Histone H3 using multiparametric (coupled to DNA content analysis) flow cytometry [11]. In mice oral administration of AMG 900 inhibits phosphorylation of histone H3 in tumors and bone marrow in a concentration- and dose-dependent manner. In order to further refine this aurora-B biochemical biomarker, we evaluated the effects of AMG 900 on p-Histone H3 in human tumor xenografts, mouse skin, and mouse bone marrow tissues using both flow cytometry (FCM) and automated laser scanning cytometry (LSC) detection platforms. Because these preclinical studies had the potential to inform our AMG 900 clinical biomarker program, we monitored PD assay parameters (e.g. sensitivity, precision, and specificity) in tumor bearing mice (n = 10 per group). We demonstrate that AMG 900 inhibits p-Histone H3 in tumors and surrogate tissues. These findings support the use of p-Histone H3 as a biomarker to determine plasma levels of AMG 900 required to inhibit aurora-B activity. Notably, mouse skin was not a particularly sensitive surrogate tissue for assessing tumor PD effects as compared to the tumor or the bone marrow. Therefore, we evaluated a translational approach to study on-target PD effects of AMG 900 using a more relevant biopsy method, fine needle aspirates (FNAs) [12-14] to directly measure AMG 900 activity in the tumor itself. For this proof-of-concept translational study, we demonstrate that treatment with AMG 900 significantly reduced p-Histone H3 (>99% inhibition, *p* <0.0001) in COLO 205 tumor FNAs, suggesting that FNA biopsies may be a viable approach for assessing AMG 900 PD effects in the clinic*.* Lastly, we illustrate that this LSC-based approach can detect p-Histone H3 positive cells using mock FNAs from primary human breast tumor tissue.

## Methods

### Small molecules

AMG 900 N-(4-((3-(2-amino-4-pyrimidinyl)-2-pyridinyl)oxy)phenyl)-4-(4-methyl-2-thienyl)-1-phthalazinamine) was synthesized at Amgen (WO2007087276). For *in vivo* studies, AMG 900 was formulated as a suspension in 2% HPMC, 1% Tween-80, at pH 2.2. Nocodazole was procured from Sigma-Aldrich.

### Animal and cell line information

Female athymic nude mice (Harlan Sprague Dawley) of approximately 14 weeks of age were housed five per sterilized filter-capped cages and maintained under aseptic and pathogen-free conditions. The animal holding room provided 12 hours of alternating light and dark cycles and met the standards of the Association for Assessment and Accreditation of Laboratory Animal Care specifications. Commercial rodent chow, filter-purified tap water was offered *ad libitum*, and nutritional supplements were provided daily. All animal studies were performed in accordance with Amgen’s Institutional Animal Care and Use Committee and U.S. Department of Agriculture Regulations. All drugs were administered based on the individual body weight of each mouse. Genetically authenticated COLO 205 cells (human colorectal adenocarcinoma cell line) were procured from American Type Culture Collection (ATCC). Cells were cultured in media supplemented with 10% FBS using conditions specified by ATCC.

### COLO 205 tumor xenografts

Mice were injected subcutaneously with 2 ×10^6^ COLO 205 cells in 100 μL of 50% matrigel (BD Biosciences). Mice with established tumors (approximately 200 mm^3^) were assigned into experimental groups (n = 10 per group) and administered a single oral dose of vehicle or AMG 900 at 3.75, 7.5, and 15 mg/kg. Three hours after treatment tissue specimens (bone marrow, tumor, and skin) were collected from individual mice for pharmacodynamic and histological analysis. Blood plasma samples (50 μL) were collected from individual mice to determine the concentration of AMG 900 using quantitative methods previously described [15]. Excised tumors were divided in half for parallel flow and imaging based cytometric analyses.

### COLO 205 tumor and bone marrow p-Histone H3 assessment by FCM

Excised tumors were immediately minced into fine pieces with a razor blade and transferred into 10 mL of cell dissociation buffer [1XHBSS (Invitrogen), 0.1 mg/mL Collagenase-V (Sigma-Aldrich), 0.25 mg/mL Collagenase-XI (Sigma-Aldrich), 1% Dispase II (Sigma-Aldrich) and 25 units/mL RNase-free DNase I (Roche)] in a 50 mL conical tube with a small magnetic stir bar. Tubes were inverted and placed on a magnetic stir plate for 30 minutes at 37°C with constant stirring to facilitate dissociation into a single-cell suspension, and passed through 70 micron nylon filter (BD Falcon) and centrifuged at 2000 rpm for four minutes at 18°C. Tumor cell pellets were washed in 1 mL of Versene (Invitrogen) and centrifuged at 2000 rpm for one minute at room temperature. To finish, cell pellets were fixed in ice-cold 90% methanol and stored overnight at −20°C before immunocytochemical staining. To isolate mouse bone marrow (BM) cells, trimmed femur bone were flushed with 1 mL of BM harvest buffer [1X HBSS, 1% BSA (Bovine Albumin Path-O-Cyte 5, ICN Biomedicals)] using 1 mL syringe, cells were centrifuged at 2000 rpm for one minute at 18°C, fixed in ice-cold 90% methanol and stored overnight at −20°C before immunocytochemical staining. Fixed tumor and bone marrow cells (~1 ×10^6^ cells/tube) were centrifuged at 2000 rpm for four minutes and washed/permeabilized once with Wash Buffer [1X PBS (Invitrogen), 1% BSA, 0.2% Triton X-100 (Sigma-Aldrich)]. Bone marrow cells were further processed by incubating the cells with 100 μL of Acid Buffer (2 N HCl, 0.5% Triton X-100 in distilled water) for 45 minutes at room temperature. Cells were washed thrice in Wash Buffer. Next, cells were stained with 2 μg/mL of anti-p-Histone H3 serine-10 antibody (Millipore) and 30 μL per tube anti-cytokeratin-FITC antibody (COLO 205 tumors only, Clone 5D3, cytokeratin (8/18), Vector Labs) for 1.5 hours at room temperature in the dark. After centrifugation, cells were washed, and then stained with 1.5 μg/mL detection antibody (anti-rabbit IgG alexa-647 second antibody, Invitrogen) for 45 minutes at room temperature in the dark. Stained cells were centrifuged, washed and counterstained with 200 μL of propidium iodide (PI) supplemented with RNAse I (BD Biosciences) for 30 minutes at room temperature in the dark, passed through 35-micron nylon mesh filter and transferred into 5 mL FACS tubes (Falcon) containing 400 μL of PI. Data was acquired using a LSRII flow cytometer running FACSDiva software (BD Biosciences Immunocytometry Systems). A primary gating strategy was based on DNA content (double discrimination) and a nested gate on cytokeratin fluorescence intensity (COLO 205 tumors only). A threshold gate was then applied on the G_2_M cell population (4 N DNA content) and its corresponding p-Histone H3 subpopulation (see Additional file 1: Figure S1). The total percentage of p-Histone H3 positive events in G_2_M was determined for each bone marrow and tumor sample.

### COLO 205 tumor and skin p-Histone H3 assessment by LSC

As described above tumor and mouse tissues were collected from each animal for p-Histone H3 analysis by LSC including: half of each excised tumor, two skin punch biopsies (left and right flanks), and small intestine as an internal proliferating tissue control. Tumor and tissues were sectioned and then arrayed onto glass microscope slides. After deparaffinization, rehydration, antigen retrieval, and overnight blocking, slides were immunocytochemically stained on an automated LabVision Autostainer 720™ [16]. Briefly, following five minutes of permeabilization with 0.2% Triton X-100 in 1X PBS, the slides were incubated in blocking/staining buffer (1X PBS, 0.5% BSA, PBS/BSA) for ten minutes at room temperature. Next, slides were incubated in 2 μg/mL of anti-p-Histone H3 antibody in PBS/BSA for 45 minutes. Slides were thoroughly rinsed in PBS/BSA and stained with a secondary detection antibody (2 μg/mL anti-rabbit IgG alexa-647) in PBS/BSA for 45 minutes. Slides were rinsed for ten minutes in PBS/BSA and counterstained with the DNA dye DAPI (4′,6-Diamidino-2 Phenylindole, Dihydrochloride) and mounted with glass cover slips containing anti-fade reagent (Invitrogen). Data was acquired using an iCys Laser Scanning Cytometer (CompuCyte) and its affiliated iGeneration software (version 3.2.4/3.3.1). Scanned images were obtained using a 20× objective to quantify the fluorescent intensity and laser absorbance (light loss) signals for each specimen. The tissue sections were scored using a two-step LSC-protocol that automates the quantitation of p-Histone H3 positive events (counts) as a function of tissue area (mm^2^). P-Histone H3 positive events were identified (contoured) using long-red channel in high resolution scan mode. Topological tissue maps were generated during data analysis by overlaying a lattice of small circles (10 micron radius) on top of the field images and mapping the density of green autofluorescence or blue fluorescence (DNA counterstain) inside each circle as a function of its x/y coordinates. We mapped the p-Histone H3-positive cell positions by plotting all long-red contoured objects as a function of their x/y coordinates (see Additional file 2: Figure S2).

### COLO 205 tumor fine needle aspirates (FNAs) p-Histone H3 assessment by LSC

Mice with established COLO 205 tumors (approximately 200 mm^3^) were administered a single oral dose of vehicle alone or AMG 900 at 15 mg/kg. At three hours, tumor aspirates (three replicates per tumor) were collected for pharmacodynamic evaluation. Tumor aspirates were collected by inserting a 25-gauge needle (BD Biosciences) through a small incision in the skin surrounding the tumor in a predetermined and consistent punch pattern, and then expelled into 2% paraformaldehyde. Fixed cell suspensions were spotted on microscope slides using cytospin chambers and centrifugation (Shandon). As positive controls, COLO 205 cells in culture were treated with DMSO or 100 ng/mL nocodazole for 16 hours and processed in the same manner as described for xenograft tumors. Slides were immunocytochemically stained for p-Histone H3 (alexa-647, CST) and counterstained with DAP, as above. Images were acquired using an iCYS laser scanning cytometry equipped with a 40× objective (high resolution scan). Object segmentation was based on DNA content (cell-cycle phase) and p-Histone H3 positive objects were verified by relocating images of mitotic object into galleries. The percentage of p-Histone H3 positive objects in G_2_M was determined for each FNA sample.

### Primary breast tumor fine needle aspirates p-Histone H3 assessment by LSC

Freshly resected human breast tumors were obtained from Asterand (Detroit, MI) and Bio-Options (Fullerton, CA) within 18 hours of surgery. Mock FNAs were performed on breast tumors using a 22-gauge × 6-inch Chiba needle (Becton Dickinson) attached to a 12-mL syringe, and samples were deposited into a 2-mL tube (Sarstedt) containing 1.5 mL of 4% paraformaldehyde. Tubes were gently inverted five times to adequately suspend the cells, and samples were stored at 4°C. Fixed cell suspensions were spotted on microscope slides using cytospin chambers and centrifugation (Shandon). Slides were immunocytochemically stained with directly conjugated antibodies specific for EpCAM (epithelial tumor marker, alexa-488, BD Biosciences) and p-Histone H3 (alexa-647, CST) and counterstained with DAPI, as above. Images were acquired using an iCYS laser scanning cytometry equipped with a 40× objective (high resolution scan). Object segmentation was based on DNA content (cell-cycle phase), and anti-EpCAM positivity. The authenticity of the p-Histone H3 positive objects was verified by relocating images of mitotic object into galleries.

### Statistical analysis

**Figure 1:**
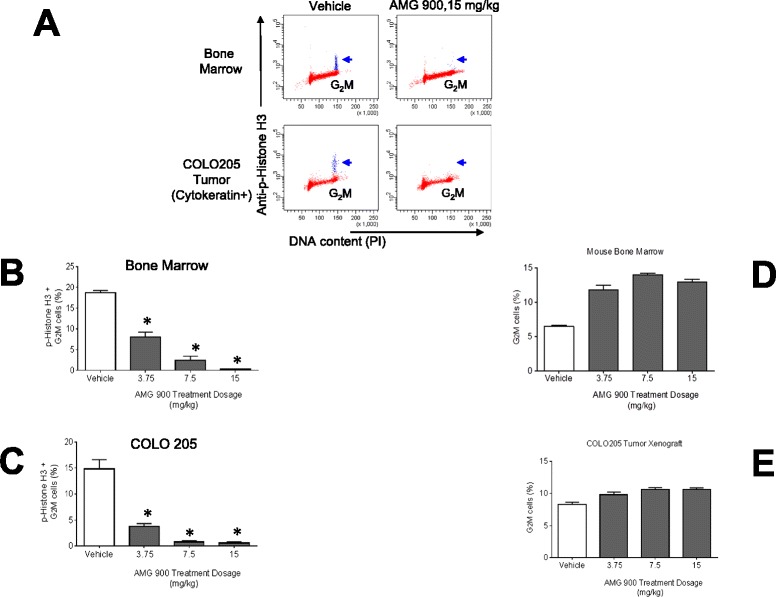
**AMG 900 inhibits p-Histone H3 and increases the percentage of G**_**2**_ **M cells in a dose-dependent manner in COLO 205 tumors and mouse bone marrow measured by Flow Cytometry (FCM).** Mice bearing established tumors were orally administered a single dose of vehicle alone or AMG 900 at 3.75, 7.5, or 15 mg/kg. Bone marrow and tumor specimens were collected three hours after treatment (n = 10 per treatment group) and processed for p-Histone H3 and DNA content analysis by FCM. **(A)** Representative cell cycle profiles of bone marrow (*upper panel*) and tumor (*lower panel*, COLO 205 tumor cells were identified using an anti-cytokeratin antibody). AMG 900 treatment decreases the p-Histone H3 positive cell population in G_2_M detectable in the vehicle-treated control (*blue, arrow*). Column graphs represent the percentage of p-Histone H3 positive G_2_M cells **(B and** **C)** and G_2_M cells **(D and** **E)** for each treatment group (mean + SE). Statistical significance was determined by comparing the individual AMG 900 treatment groups with vehicle-treated control (*P <0.0001).

**Figure 2:**
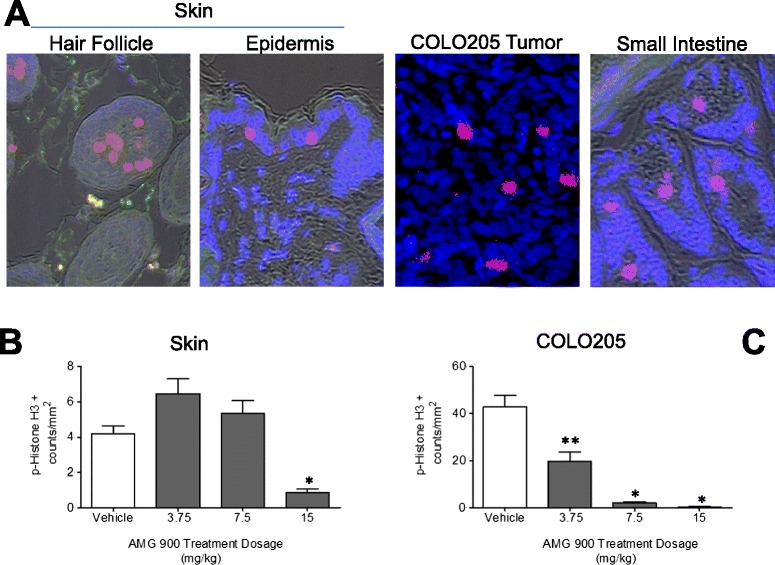
**AMG 900 inhibits p-Histone H3 in COLO 205 tumors and mouse skin (hair follicle, epidermis) in a dose-dependent manner measured by Laser Scanning Cytometry (LSC).** Mice bearing established tumors were orally administered a single dose of vehicle alone or AMG 900 (as described in Figure 1). Mouse skin specimens (a right- and left-flank punch biopsy from each animal) were collected three hours after treatment (n = 10 per treatment group) and processed for p-Histone H3 analysis by LSC. Small intestine was also collected as a positive staining control for p-Histone H3. Images were captured on a LSC (iCys, CompuCyte) using a 20× objective. See *Materials and Methods Section* for tissue and tumor section immunofluorescence staining procedure and Additional file 2 Figure S2A for the random sampling contour strategy. **(A)** Representative scan fields of mouse tissues (skin, hair follicle, small intestine) and COLO 205 tumor showing p-Histone H3 positive objects (*red* or *pink*). Nuclei were counterstained with Hoechst 33342 (*blue*). An average p-Histone H3 object count (per mm^2^) was determined from three scan fields for each tissue section. Column graphs represent p-Histone H3 positive object for skin **(B)** and COLO 205 tumor **(C)** (mean + SE). Statistically significant inhibition of p-Histone H3 compared with vehicle-treated control (*P <0.0001; **P =0.0005).

The effects on p-Histone H3 by AMG 900 and vehicle-control treatment groups were compared using either Oneway ANOVA followed by Dunnett’s post hoc analysis (Figure 1) or using ANOVA followed by Bonferroni Dunnett’s post hoc analysis (Figure 2). Data were represented as the standard error of the mean (±SEM) for each treatment group. Differences were considered significant at a *P* value of <0.05. Graphing and linear regression analysis was performed using GraphPad Prism software.

## Results

### AMG 900 inhibits p-Histone H3 in COLO 205 human tumor xenografts and mouse surrogate tissues as measured by FCM and LSC

The primary objective of building a robust PK-PD relationship is to directly link drug exposure (e.g. dosage, plasma concentration) to PD effect in desired target tissue(s). Aurora-B kinase activity is essential for cell division [17]; therefore, normal and tumor proliferating tissues are potentially suitable for assessing its enzymatic activity. In order to examine the effects of aurora-B inhibition by AMG 900 in proliferating tissues (tumor, bone marrow, and skin), we evaluated FCM and LSC cytometric approaches to determine the level of p-Histone H3 in single-cell tumor suspensions and tissue sections. Mice with established COLO 205 tumors were administered a single oral dose of vehicle alone or AMG 900 at 15, 7.5, and 3.75 mg/kg (n = 10 per group). We chose a three hour post-dose time point for our PD assessment [8]. As shown in Figure 1A, treatment with AMG 900 at 15 mg/kg significantly inhibited p-Histone H3 in the G_2_M cell population in mouse bone marrow (*upper panel*) and cytokeratin positive COLO 205 tumor (*lower panel*) compared with vehicle-treated controls. Figure 1B and C show the mean level of p-Histone H3 inhibition by AMG 900 in bone marrow and tumor, respectively. Oral administration of AMG 900 at 7.5 and 15 mg/kg resulted in near maximal inhibition of p-Histone H3 (>85%, p <0.0001), whereas the lower dose of 3.75 mg/kg showed partial but significant inhibition of p-Histone H3 (>57%, p <0.0001). We also observed a corresponding increase in total G_2_M cells in both bone marrow and tumor in mice treated with AMG 900 (Figure 1D and E). Next we tested the feasibility of using skin as a surrogate tissue to assess the overall PD impact of AMG 900. Hair follicle, skin, and oral buccal mucosa provide a non-invasive source of proliferating normal cells (potential alternative to more invasive bone marrow aspiration) that may prove useful for establishing PK-PD relationship. This approach was assessed in mice by comparing the levels of p-Histone H3 *in situ*, in xenograft sections, in parallel with the levels of p-Histone H3 in skin punch biopsy sections prepared from both flanks of the mouse and evaluated by LSC. Sections of formalin-fixed paraffin embedded xenograft tumors and skin biopsies were immunocytochemically stained for p-Histone H3 as described in the materials and methods section. We used a stereological sampling methodology to quantify the number of p-Histone H3 positive objects and reported the data as a function of area (counts per mm^2^) [18] (Additional file 2: Figure S2A). As shown in Figure 2A, p-Histone H3 positive nuclei were detectable in both mouse surrogate tissues (skin, hair follicle, small intestine) and COLO 205 xenograft tumor. Staining with anti-p-Histone H3 antibody produced a robust fluorescent signal with low non-specific background. We also confirmed the mitotic specificity of p-Histone H3 positive objects using the relocation feature on the LSC; this creates an image gallery of contoured objects (Additional file 2: Figure S2B).

Having established the methods for detecting robust p-Histone H3 staining using tissue and tumor xenograft sections, we next evaluated the tumor and tissue sections treated with vehicle alone or AMG 900 as described above. Mirroring our FCM based analysis, we detected a marked decrease in p-Histone H3 positive objects in COLO 205 tumors treated with AMG 900 at 15 and 7.5 mg/kg doses, whereas the lower dose of 3.75 mg/kg showed partial inhibition of p-Histone H3 (p ≤0.0005) (Figure 2C). The PD response in tumor measured by FCM and LSC were remarkably similar for AMG 900 dose groups (Figures 1C and 2C), suggesting that both approaches are suitable for measuring p-Histone H3 in tumors. The level of p-Histone H3 positive objects observed in skin for the vehicle-control group was markedly lower compared with tumor (roughly 10-fold lower), whereas the fluorescence intensity of p-Histone H3 objects was similar across skin and tumor. This lower frequency of mitotic objects observed in skin tissue is likely due to the lower frequency of actively cycling cells in the basal layer of the epidermis compared to more proliferative human tumor cells from xenograft tissue. Only mice treated with AMG 900 at 15 mg/kg showed a statistically significant suppression in p-Histone H3 (p <0.0001), indicating that skin may be an unacceptable tissue for monitoring PD response compared with either bone marrow or tumor tissue sampling (Figure 2B).

**Figure 3:**
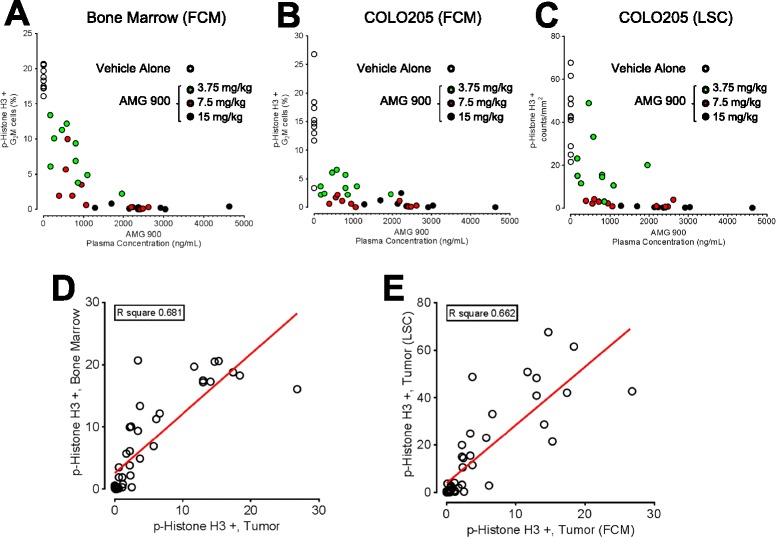
**Mouse plasma concentration of AMG 900 correlates with the degree of p-Histone H3 suppression in both COLO 205 tumor and mouse bone marrow tissues.** Mice bearing established tumors were orally administered a single dose of vehicle alone or AMG 900 (as described in Figure 1). Mouse plasma was collected three hours after treatment (n = 10 per treatment group). Scatter plots were generated using the observed percentage of p-Histone H3 positive G_2_M cells for bone marrow **(A)** and COLO 205 tumor **(B and** **C)** and plasma concentration of AMG 900 (ng/mL) from individual mice. Treatment groups are indicated (○ vehicle, “green circle symbol” 3.75, “red circle symbol” 7.5, and ● 15 mg/kg). Regression analysis was used to assess linearity of p-Histone H3 response relationship across tissue sets **(D)** FCM bone marrow versus tumor, **(E)** FCM tumor versus LSC tumor. The correlation coefficient (R-squared value) and fitted line (*red*) are shown for each plot.

In order to assess the PK-PD relationship, AMG 900 plasma levels were determined as described elsewhere [19] for all three dosage groups and correlated with bone marrow (FCM) and COLO 205 tumor PD response (FCM and LSC). We included the vehicle treated group as a PD baseline control. The PK-PD data presented in Figure 3A-C shows the level of p-Histone H3 suppression largely correlates with the concentration of AMG 900 in mouse plasma using both FCM and LSC platforms. The majority of test samples with >1000 ng/mL (2 μM) plasma concentrations of AMG 900 were associated with complete inhibition of aurora-B activity as measured by the decrease in p-Histone H3. In contrast, partial inhibition of p-Histone H3 by AMG 900 (3.75 mg/kg group) was mostly associated with lower concentrations of drug (range 300 to 1000 ng/mL). Linear regression analysis of individual paired PD responses were mostly concordant across tissues [FCM, bone marrow versus tumor (R^2^ = 0.681, Figure 3D); and FCM versus LSC, tumor (R^2^ = 0.662, Figure 3E)]. Together, these data suggests that bone marrow is an acceptable PD surrogate for tumor and that both FCM and LSC approaches are sensitive and complimentary methods of measuring aurora-B activity *in vivo.*

### AMG 900 inhibits p-Histone H3 in tumor cells using fine-needle aspirate (FNA) biopsies

**Figure 4:**
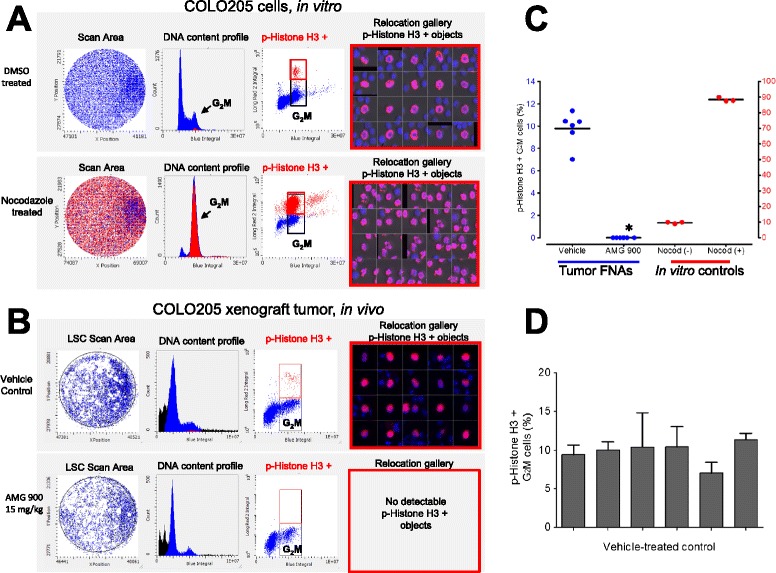
**AMG 900 inhibits p-Histone H3 in COLO 205 tumor cells using fine-needle aspirate (FNA) biopsies. (A)** LSC based *in vitro* cell cycle assessment of COLO 205 tumor cells treated with DMSO or 100 ng/mL nocodazole. Cytospin deposited cells were immunostained with an anti-p-Histone H3 antibody and counterstained with DAPI. Plots represent the cell cycle profile indicating G_2_M (*arrow*), and p-Histone H3 (*red*) subpopulations. Relocation gallery of representative scanned images of p-Histone H3 positive cells in mitosis. **(B and** **C)** Mice bearing established COLO 205 tumors were orally administered a single dose of vehicle alone or AMG 900 at 15 mg/kg. Tumor FNA punch biopsies (n = 3 per tumor) were collected three hours after treatment (n = 6 per treatment group) and processed for p-Histone H3 and DNA content analysis by LSC. **(B)** Representative tumor FNA cell cycle profiles of vehicle (*upper panel)* and AMG 900 (*lower panel)* treatment groups. AMG 900 treatment completely abolished the p-Histone H3 positive cell population in G_2_M detectable in the vehicle-treated control (*red*). **(C)** Scatter plot represents the individual and mean percentage of p-Histone H3 positive G_2_M cells for each treatment group. Statistically significant inhibition of p-Histone H3 compared with vehicle-treated control (*P <0.0001). COLO 205 tumor cells were treated with 100 ng/mL nocodazole (Nocod +) or DMSO (Nocod -) as p-Histone H3 staining controls. **(D)** Column graph represents vehicle-treated control group inter-(6 tumors) and intra-tumor FNA (3 per tumor) variation in the percentage of p-Histone H3 positive G_2_M cells (mean + SE).

Fine-needle aspiration is an inexpensive biopsy technique used routinely for diagnosis [20,21] and has recently been adapted for immunocytochemical analysis of signal transduction pathways [12,13]. The LSC cytometric platform is well-suited for FNA sample analysis: 1) it is capable of assessing small numbers of cells from complex tissues such as tumor; 2) it retains the overall tumor phenotype [12-14,22-25] Additional file 3: Figure S3A. As shown in Figure 4A, COLO 205 tumor cells (in culture) can be identified in each phase of the cell cycle (G_1_, S, G_2_M), with a subpopulation of p-Histone H3 positive in G_2_M with distinct condensed mitotic nuclei. As anticipated, treatment with nocadozole (microtubule-targeting agent), markedly increased the mitotic cell fraction of p-Histone H3 positive objects. Cytometric quantitation of the percentage of p-Histone H3 positive objects was performed by using DNA content to restrict analysis to single cells in the G_2_M phase.

**Figure 5:**
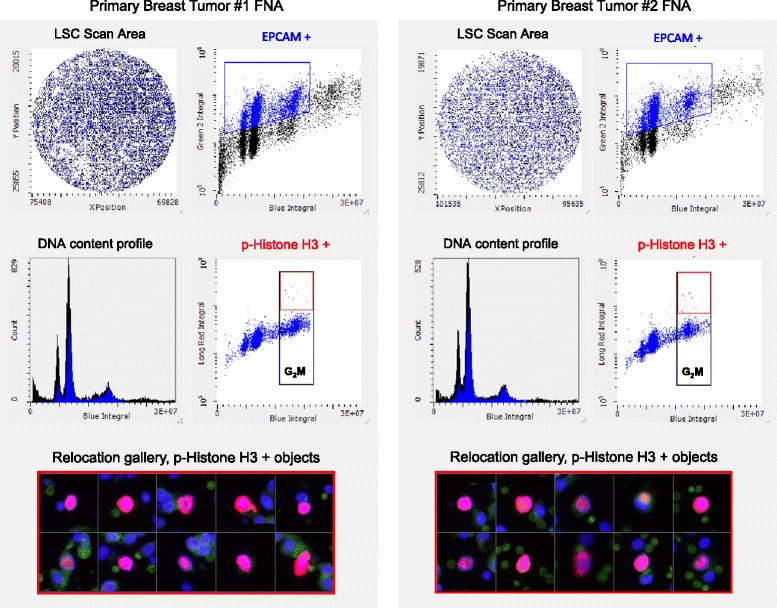
**Detection of p-Histone H3 using primary human breast tumor FNA biopsies by LSC.** Two mock FNA punch biopsies were collected from the same fresh primary breast tumor tissue. Cytospin deposited cells were immunostained with anti-EpCAM antibody (epithelial cell specific marker) and anti-p-Histone H3 antibodies and counterstained with DAPI. Representative cell cycle profiles of two FNAs showing EpCAM positive (*blue*) tumor cells with a subpopulation of p-Histone H3 positive G_2_M cells (*red*). Relocation gallery showing p-Histone H3 positive objects.

As a proof-of-concept experiment designed to support the use of FNA sampling, mice with established COLO 205 tumors (n = 6 per group) were administered a single oral dose of vehicle alone or AMG 900 at 15 mg/kg. Tumor FNAs (n = 3 per tumor) were collected three hours after treatment and immediately fixed and processed for immunofluorescence staining with an anti-p-Histone H3 antibody. Mice treated with AMG 900 showed near-complete inhibition of p-Histone H3 in COLO 205 tumor FNAs compared with vehicle-treated control (Figure 4B and C). Next, we obtained fresh primary breast tumor tissue to evaluate the ability to detect p-Histone H3 positive cells using the same FNA sampling approach. To restrict our analysis to tumor cells, the FNAs were co-stained with an anti-EpCAM antibody (epithelial cell specific marker). As shown in Figure 5 (and Additional file 3: Figure S3B), the EpCAM positive p-Histone H3 G_2_M tumor cell population was readily detectable in two mock FNA samples taken from the same primary breast tumor. The authenticity of these rare (<1%) p-Histone H3 objects was confirmed using the relocation feature on the LSC (Figure 5, *lower panel*). The frequency of p-Histone H3 positive cells in this primary breast tumor was markedly lower compared to COLO 205 tumor xenografts. Taken together, these data indicate that testing FNAs for p-Histone H3 immunoreactivity in tumor cells is a viable approach to survey the PD activity of AMG 900 in target tissues.

## Discussion

Aurora kinases have emerged as attractive anticancer targets because of their key roles in regulating mitotic progression and associated aberrant expression in a number of human malignancies [26-33]. Anti-mitotic drugs that target the microtubules (e.g. taxanes and vinca alkaloids) have certain limitations that include MDR and peripheral neuropathy [5,30]. To optimize the clinical development of these promising drug candidates, efforts have been made to identify and validate potential biomarkers, including pharmacodynamic assessments in normal blood cells, skin or/and tumor biopsies (reviewed in Perez-Fidalgo et al. [27]).

The present preclinical report describes the efforts to develop PD biomarkers that can be effectively translated to the clinical setting for AMG 900. Specifically, our studies address whether aurora-B activity is modulated in tumor and surrogate tissues from animals treated with AMG 900. Aurora-B plays a number of key roles in mitotic processes, including phosphorylation of histone H3 (necessary for chromosome condensation) and therefore, measuring inhibition of phosphorylation of Histone H3 at serine-10 represents a direct readout of PD impact [10]. Exploratory biomarker studies with different aurora kinase inhibitor candidates in a clinical setting have been used for both tumor and skin biopsies [7,34].

We investigated the feasibility of using mouse surrogate tissues (bone marrow and skin) to sample the overall PD impact of AMG 900 and directly compared the effects to the levels of p-Histone H3 in tumor sections. The baseline numbers of p-Histone H3 counts/mm^2^ in skin tissue sections were markedly lower compared to the tumor sections, whereas the immunoreactivity (MFI signal) was similar. These data indicate that skin tissue has a smaller fraction of actively cycling cells. Surprisingly, only the higher dose of AMG 900 (15 mg/kg) effectively suppressed p-Histone H3 in the skin, whereas the middle and lower doses of compound failed to show a PD response, suggesting that skin may lack sufficient sensitivity to use as a surrogate tissue readout. In contrast, measuring suppression of aurora-B activity in tumor and bone marrow, provided similar levels of p-Histone H3 inhibition, which may provide the translational possibility of detecting early evidence of drug activity prior to clinical signs of tumor regression. One possible explanation for the absence of a PD response (at the lower doses of AMG 900) in skin is differences in drug distribution in specific tissues, which was not determined in this study.

We next adapted our sensitive, specific, and quantitative PD assay for use with a non-invasive fine-needle aspiration sampling technique using tumor xenograft tissues. Our PD results obtained using FNAs was highly concordant with dissociation or sectioned tumor xenograft tissue, suggesting that FNAs may be a feasible approach for measuring on-target aurora-B inhibition in tissue specimens in the clinic. Moreover, there was a clear correlation between AMG 900 plasma exposure and inhibition of p-Histone H3 in tumor cells. We confirmed the LSC cytometric platform was ideal for FNA sample analysis, where it was capable of assessing small numbers of mitotic cells from complex tissues such as tumor FNAs. For example, the PD assessment of a freshly resected breast tumor biopsy using a mock FNA approach showed only a small fraction (<1%) of EpCAM & p-Histone H3 positive objects. Because of this limited number of tumor cells, it will be critically important to have high quality tumor FNA samples to obtain acceptable PD results using LSC analysis. Previously, we described pre-clinical and clinical implementation of another cytometric imaging assay that measures activated caspase-3 using tumor FNAs [14,35]. It is important to note that phosphorylation of Histone H3 can occur in all cell cycle phases during chromatin condensation events such as premature chromosome condensation [36], and in interphase during chromatin changes accompanying monocytic lineage differentiation [37]. Our approach restricted analysis to G_2_M cell population, therefore it is unlikely that phosphorylation of Histone H3 during interphase would interfere with monitoring aurora-B activity in mitosis.

## Conclusions

The present preclinical report describes the efforts to effectively develop pharmacodynamic biomarkers that can be translated to the clinical setting for AMG 900, a small-molecule inhibitor of pan-aurora kinases. p-Histone H3 measurement is a potentially useful biomarker in clinical studies to determine plasma levels of AMG 900 required to inhibit aurora-B activity. FNA biopsies may be a viable approach for assessing AMG 900 PD effects in the clinic.

## Additional files

Additional file 1: Figure S1. Flow cytometry gating scheme. Representative bone marrow **(A)** and COLO 205 tumor **(B)** processed for p-Histone H3 and DNA content analysis by FCM. Representative left plots to identify single events and cell cycle vs p-Histone H3 profiles of bone marrow (*upper panel*) and tumor (*lower panel*, COLO 205 tumor cells identified using an anti-cytokeratin antibody). p-Histone H3 positive cell population in G_2_M (*blue*). Additional file 2: Figure S2. LSC segmentation scheme COLO205 tumor sections. Representative tumor xenograft section stained with DAPI and anti-p-Histone H3 as assessed by laser scanning cytometry. **(A)** Image analysis involved stereologic sampling of fields with a lattice of circular contours to measure tumor area and cellular segmentation of positively stained anti-p-Histone H3 events. p-Histone H3 events were unambiguously labelled (left) and accurately contoured (middle). Topological tissue maps were generated during data analysis by overlying a lattice of small circles (r = 10 μm) on top of the field images (right). **(B)** Cytometric gating quantitation (left), p-Histone H3 cell positions were mapped by plotting all long red contoured events from the selected R1 gate as a function of their x/y coordinates (middle) and positive p-Histone H3 events were confirmed using image relocation galleries (right). Additional file 3: Figure S3. LSC segmentation scheme Tumor FNAs. **(A)** (U*pper panel) *Cytospun deposited COLO 205 FNAs were immunostained with an anti-p-Histone H3 antibody and counterstained with DAPI. Representative scanned image fields and cellular segmentation based on DAPI (nuclear stain) and positive p-Histone H3 events (red). *Lower panel* from left show representative cytometric gating plot to exclude aggregates and identify single events, the cell cycle profile (middle plot), and p-Histone H3 (*red*) in G_2_M cells (right plot). **(B)** Representative mock FNA from a human primary breast tumor. Cytospun deposited FNAs were immunostained with an EpCAM antibody, an anti-p-Histone H3 antibody and counterstained with DAPI. Representative scanned image fields and cellular segmentation based on DAPI (nuclear stain). *Lower panel* from left show representative cytometric gating plot to identify EpCAM positive and negative events, the cell cycle profile (middle plot) for both populations and and p-Histone H3 (*red*) in G_2_M cells of EpCAM positive subset cells (right plot).

## References

[1] Warner SL, Bearss DJ, Han H, Von Hoff DD (2003). Targeting aurora-2 kinase in cancer. Mol Cancer Ther.

[2] Gizatullin F, Yao Y, Kung V, Harding MW, Loda M, Shapiro GI (2006). The aurora kinase inhibitor VX-680 induces endoreduplication and apoptosis preferentially in cells with compromised p53-dependent postmitiotic checkpoint function. Cancer Res.

[3] Carmena M, Earnshaw WC (2003). The cellular geography of aurora kinases. Nat Rev Mol Cell Biol.

[4] Jackson JR, Patrick DR, Dar MM, Huang PS (2007). Targeting anti-mitotic therapies: can we improve on tubulin agents?. Nat Rev Cancer.

[5] Harrington EA, Bebbington D, Moore J, Rasmussen RK, Ajose-Adeogun AO, Nakayama T, Graham JA, Demur C, Hercend T, Diu-Hercend A, Su M, Golec JMC, Miller KM (2004). VX-680, a potent and selective small-molecule inhibitor of the aurora kinases, suppresses tumor growth *in vivo*. Nat Med.

[6] Carpinelli P, Ceruti R, Giorgini ML, Cappella P, Gianellini L, Croci V, Degrassi A, Texido G, Rocchetti M, Vianello P, Rusconi L, Storici P, Zugnoni P, Arrigoni C, Soncini C, Alli C, Patton V, Marsiglio A, Ballinari D, Pesenti E, Fancelli D, Moll J (2007). PHA-739358, a potent inhibitor of aurora kinases with a selective target inhibition profile relevant to cancer. Mol Cancer Ther.

[7] Wilkinson RW, Odedra R, Heaton SP, Wedge SR, Keen NJ, Crafter C, Foster JR, Brady MC, Bigley A, Brown E, Byth KF, Barrass NC, Mundt KE, Foote KM, Heron NM, Jung FH, Mortlock AA, Boyle FT, Green S (2007). AZD-1152, a selective inhibitor of aurora B kinase, inhibits human tumor xenograft growth by inducing apoptosis. Clin Cancer Res.

[8] Payton M, Bush TL, Chung G, Ziegler B, Eden P, McElroy P, Ross S, Cee VJ, Deak HL, Hodous BL, Nguyen HN, Olivieri PR, Romero K, Schenkel LB, Bak A, Stanton M, Dussault I, Patel VF, Geuns-Meyer S, Radinsky R, Kendall R (2010). Preclinical evaluation of AMG 900, a novel potent and highly selective pan-aurora kinase inhibitor with activity in taxane-resistant tumor cell lines. Cancer Res.

[9] Ajiro K, Nishimoto T (1985). Specific site of histone H3 phosphorylation related to the maintenance of premature chromosome condensation. Evidence for catalytically induced interchange of the subunits. J Biol Chem.

[10] Giet R, Glover DM (2001). Drosophila aurora B kinase is required for histone H3 phosphorylation and condensing recruitment during chromosome condensation and to organize the central spindle during cytokinesis. J Cell Biol.

[11] Juan G, Traganos F, James WM, Ray JM, Roberge M, Sauve DM, Anderson H, Darzynkiewicz Z (1998). Histone H3 phosphorylation and expression of cyclins A and B1 measured in individual cells during their progression through G_2_ and mitosis. Cytometry.

[12] Kulesza P, Eltoum IA (2007). Endoscopic ultrasound-guided fine-needle aspiration: sampling, pitfalls, and quality management. Clin Gastroenterol Hepatol.

[13] Schwock J, Ho JC, Luther E, Hedley DW, Geddie WR (2007). Measurement of signaling pathway activities in solid tumor fine-needle biopsies by slide-based cytometry. Diagn Mol Pathol.

[14] Zoog SJ, Ma C, Kaplan-Lefko PJ, Hawkins J, Moriguchi J, Zhou L, Pan Y, Hsu C-P, Friberg G, Herbst R, Hill J, Juan G (2010). Measurement of Conatumumab-induced apoptotic activity in tumors by fine needle aspirate sampling. Cytometry A.

[15] Huang L, Zheng M, Zhou Q-M, Zhang M-Y, Jia W-H, Yun J-P, Wang H-Y (2011). Identification of a gene-expression signature for predicting lymph node metastasis in patients with early stage cervical carcinoma. Cancer.

[16] Myers J (2008). A review of automated slide strainers for IHC and ISH. Med Lab Obs.

[17] Hegyi K, Mehes G (2012). Mitotic failures in cancer: Aurora B kinase and its potential role in the development of aneuploidy. Pathol Oncol Res (Review).

[18] Henriksen M, Miller B, Newmark J, Al-Kofahi Y, Holden E (2011). Laser scanning cytometry and its applications: a pioneering technology in the field of quantitative imaging cytometry. Methods Cell Biol.

[19] Huang L, Be X, Berry L, Moore E, Janosky B, Wells M, Pan W-J, Zhao Z, Lin M-H J (2011). In vitro and in vivo pharmacokinetic characterizations of AMG 900, an orally bioavailable small molecule inhibitor of aurora kinases. Xenobiotica.

[20] Boeddinghaus I, Johnson SR (2006). Serial biopsies/fine-needle aspirates and their assessment. Methods Mol Med.

[21] Saleh H, Masood S (1995). Value of ancillary studies in fine-needle aspiration biopsy. Diagn Cytopathol.

[22] Brotherick I, Shenton BK, Lennard TW (1995). Are fine-needle breast aspirates representative of the underlying solid tumour? A comparison of receptor levels, ploidy and the influence of cytokeratin gates. Br J Cancer.

[23] Nizzoli R, Bozzetti C, Naldi N, Guazzi A, Gabrielli M, Michiara M, Camisa R, Barilli A, Cocconi G (2000). Comparison of the results of immunocytochemical assays for biologic variables on preoperative fine-needle aspirates and on surgical specimens of primary breast carcinomas. Cancer.

[24] Zabaglo L, Ormerod MG, Dowsett M (2000). Measurement of markers for breast cancer in a model system using laser scanning cytometry. Cytometry.

[25] Zabaglo L, Ormerod MG, Dowsett M (2003). Measurement of proliferation marker Ki67 in breast tumour FNAs using laser scanning cytometry in comparison to conventional immunocytochemistry. Cytometry B Clin Cytom.

[26] Keen N, Taylor S (2004). Aurora-kinase inhibitors as anti-cancer agents. Nat Rev Cancer.

[27] Perez Fidalgo JA, Roda D, Rosello S, Rodriguez-Braun E, Cervantes A (2009). Aurora kinase inhibitors: a new class of drugs targeting the regulatory mitotic system. Clin Transl Oncol.

[28] Carvajal RD, Tse A, Schwartz GK (2006). Aurora kinases: new targets for cancer therapy. Clin Cancer Res.

[29] Hilton JF, Shapiro GI (2014). Aurora kinase inhibitors as an anticancer strategy. J Clin Onc.

[30] Luo J, Solimini NL, Elledge SJ (2009). Principles of cancer therapy: oncogene and non-oncogene addiction. Cell.

[31] Cohen RB, Jones SF, Arrarwal C, von Mehren M, Cheng J, Spigel DR, Greco FA, Mariani M, Rocchetti M, Ceruti R, Comis S, Laffranchi B, Moll J, Burris HA (2009). A phase I dose-escalation study of Danusertib (PHA-737358) administered as a 24-hour infusion with and without granulocyte colony-stimulating factor in a 14-day cycle in patients with advanced solid tumors. Clin Cancer Res.

[32] Chakravarty A, Shinde V, Tabernero J, Cervantes A, Cohen RB, Dees EC, Burris H, Infante JR, Macarulla T, Elez E, Andreu J, Rodriguez-Braun E, Rosello S, von Mehren M, Meropol NJ, Langer CJ, ONeal B, Bowman D, Zhang M, Danaee H, Faron-Yowe L, Gray G, Liu H, Pappas J, Silverman L, Simpson C, Stringer B, Tirrell S, Veiby OP, Venkatakrishnan K (2010). Phase I assessment of new mechanism-based pharmacodynamic biomarkers for MLN8054, a small-molecule inhibitor of aurora A kinase. Cancer Res.

[33] Gadea BB, Ruderman JV (2005). Aurora kinase inhibitor ZM447439 blocks chromosome-induced spindle assembly, the completion of chromosome condensation, and the establishment of the spindle integrity checkpoint in Xenopus egg extracts. Mol Biol Cell.

[34] Steeghs N, Eskens F, Gelderblom H, Verweij J, Nortier JWR, Ouwerkerk J, van Noort C, Mariani M, Spinelli R, Carpinelli P, Laffranchi B, de Jonge JA (2009). Phase I pharmacokinetic and pharmacodynamic study of the aurora kinase inhibitor danusertib in patients with advanced or metastatic solid tumors. J Clin Oncol.

[35] Herbst R, Kurzrock R, Hong D, Valdivieso M, Hsu C-P, Goyal L, Juan G, Hwang Y, Wong S, Hill J, Friberg G, LoRusso P (2010). A first-in-human study of conatumumab in adult patients with advanced solid tumors. Clin Cancer Res.

[36] Huang X, Kurose A, Tanaka T, Traganos F, Dai W, Darzynkiewicz Z (2006). Sequential Phosphorylation of Ser-10 on Histone H3 and Ser-139 on Histone H2AX and ATM Activation during premature chromosome condensation: relationship to cell-cycle phase and apoptosis. Cytometry A.

[37] Juan G, Traganos F, Darzynkiewicz Z (1999). Histone H3 Phosphorylation in human monocytes and during HL-60 cell differentiation. Exp Cell Res.

