# Supplementary material for: AMG 900, a potent inhibitor of aurora kinases causes pharmacodynamic changes in p-Histone H3 immunoreactivity in human tumor xenografts and proliferating mouse tissues
Source: J Transl Med. 2014 Nov 4;12:307. doi: 10.1186/s12967-014-0307-x (PMC4221688; doi:10.1186/s12967-014-0307-x)
Supplement: Additional file 1: Figure S1. — Flow cytometry gating scheme. Representative bone marrow (A) and COLO 205 tumor (B) processed for p-Histone H3 and DNA content analysis by FCM. Representative left plots to identify single events and cell cycle vs p-Histone H3 profiles of bone marrow (upper panel) and tumor (lower panel, COLO 205 tumor cells identified using an anti-cytokeratin antibody). p-Histone H3 positive cell population in G2M (blue). [file 12967_2014_307_MOESM1_ESM.pptx]

## Slide 1
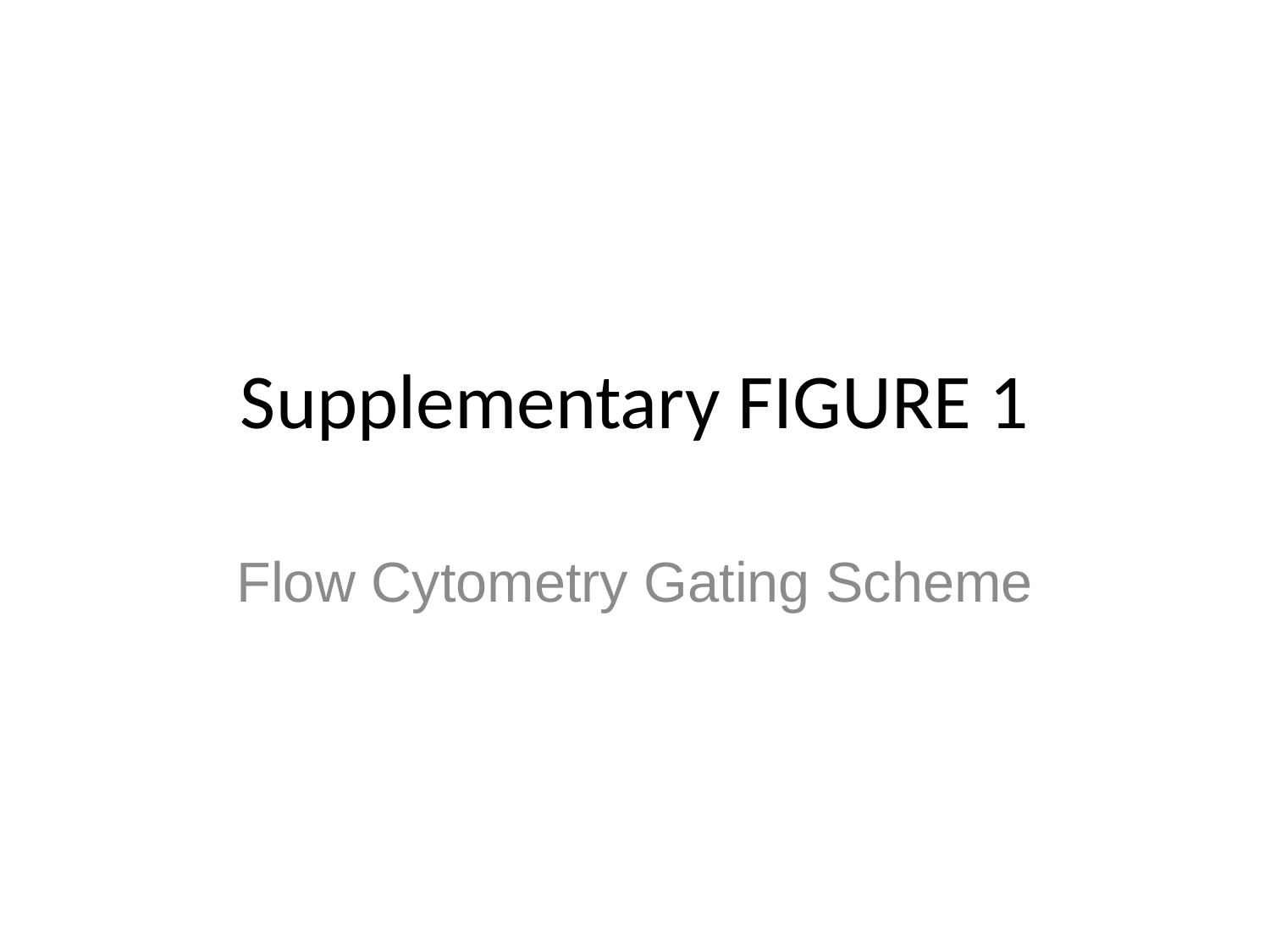

# Supplementary FIGURE 1
Flow Cytometry Gating Scheme

## Slide 2
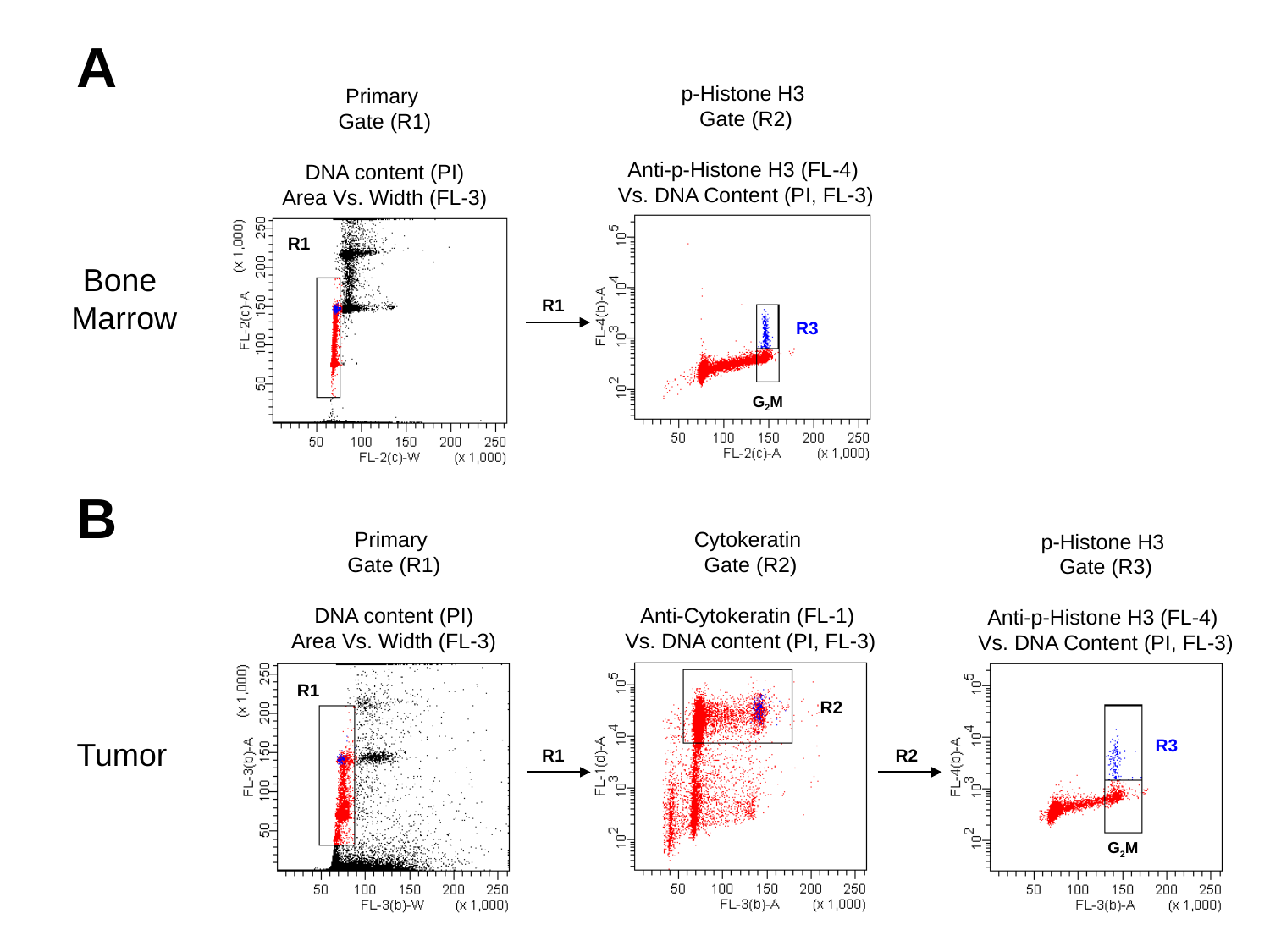

A
p-Histone H3
Gate (R2)
Anti-p-Histone H3 (FL-4)
Vs. DNA Content (PI, FL-3)
Primary
Gate (R1)
DNA content (PI)
Area Vs. Width (FL-3)
R1
Bone
Marrow
R1
R3
G2M
Primary
Gate (R1)
DNA content (PI)
Area Vs. Width (FL-3)
Cytokeratin
Gate (R2)
Anti-Cytokeratin (FL-1)
Vs. DNA content (PI, FL-3)
p-Histone H3
Gate (R3)
Anti-p-Histone H3 (FL-4)
Vs. DNA Content (PI, FL-3)
R1
R2
R3
Tumor
R1
R2
G2M
B
