# Supplementary material for: AMG 900, a potent inhibitor of aurora kinases causes pharmacodynamic changes in p-Histone H3 immunoreactivity in human tumor xenografts and proliferating mouse tissues
Source: J Transl Med. 2014 Nov 4;12:307. doi: 10.1186/s12967-014-0307-x (PMC4221688; doi:10.1186/s12967-014-0307-x)
Supplement: Additional file 2: Figure S2. — LSC segmentation scheme COLO205 tumor sections. Representative tumor xenograft section stained with DAPI and anti-p-Histone H3 as assessed by laser scanning cytometry. (A) Image analysis involved stereologic sampling of fields with a lattice of circular contours to measure tumor area and cellular segmentation of positively stained anti-p-Histone H3 events. p-Histone H3 events were unambiguously labelled (left) and accurately contoured (middle). Topological tissue maps were generated during data analysis by overlying a lattice of small circles (r = 10 μm) on top of the field images (right). (B) Cytometric gating quantitation (left), p-Histone H3 cell positions were mapped by plotting all long red contoured events from the selected R1 gate as a function of their x/y coordinates (middle) and positive p-Histone H3 events were confirmed using image relocation galleries (right). [file 12967_2014_307_MOESM2_ESM.pptx]

## Slide 1
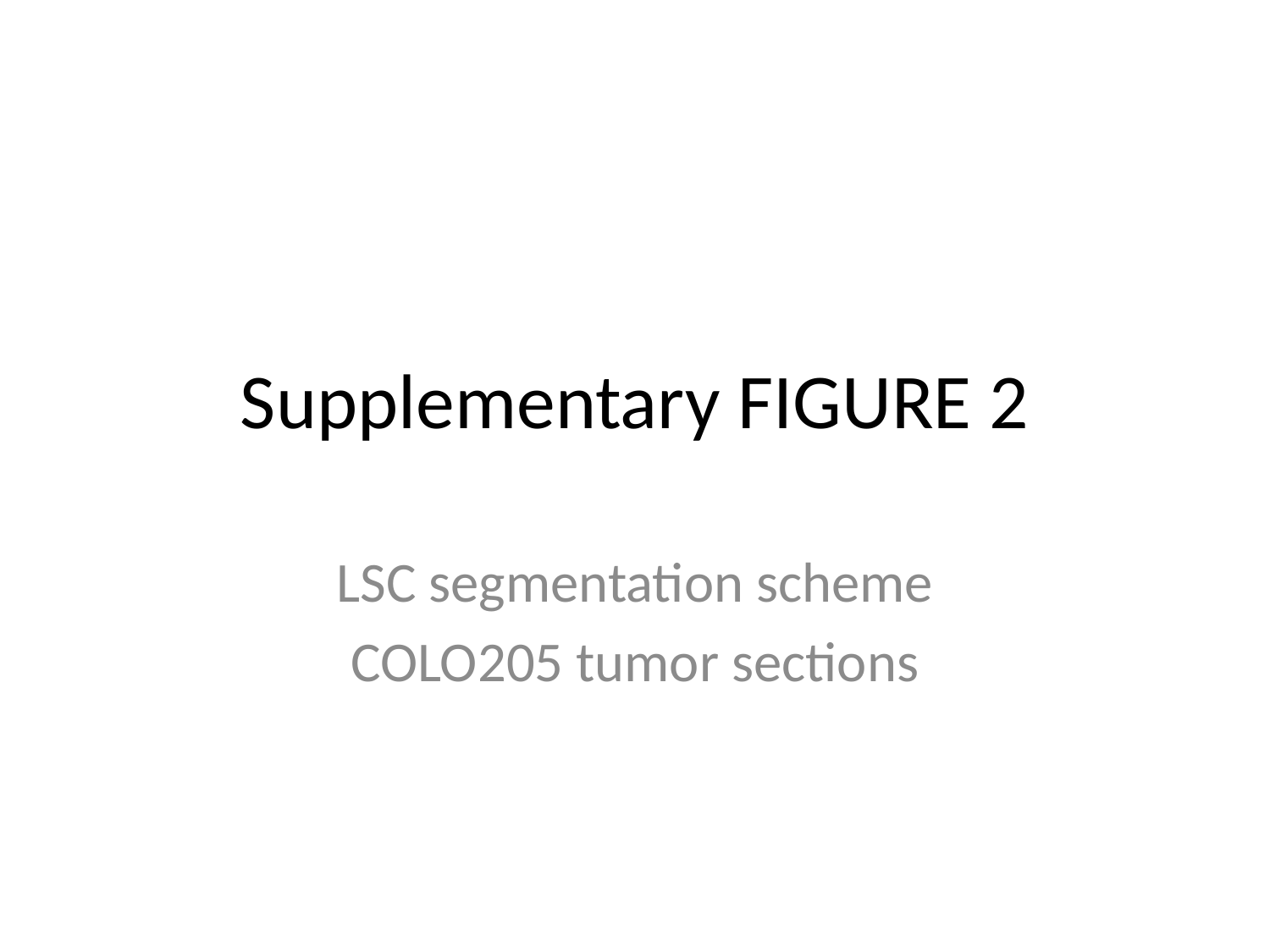

# Supplementary FIGURE 2
LSC segmentation scheme
COLO205 tumor sections

## Slide 2
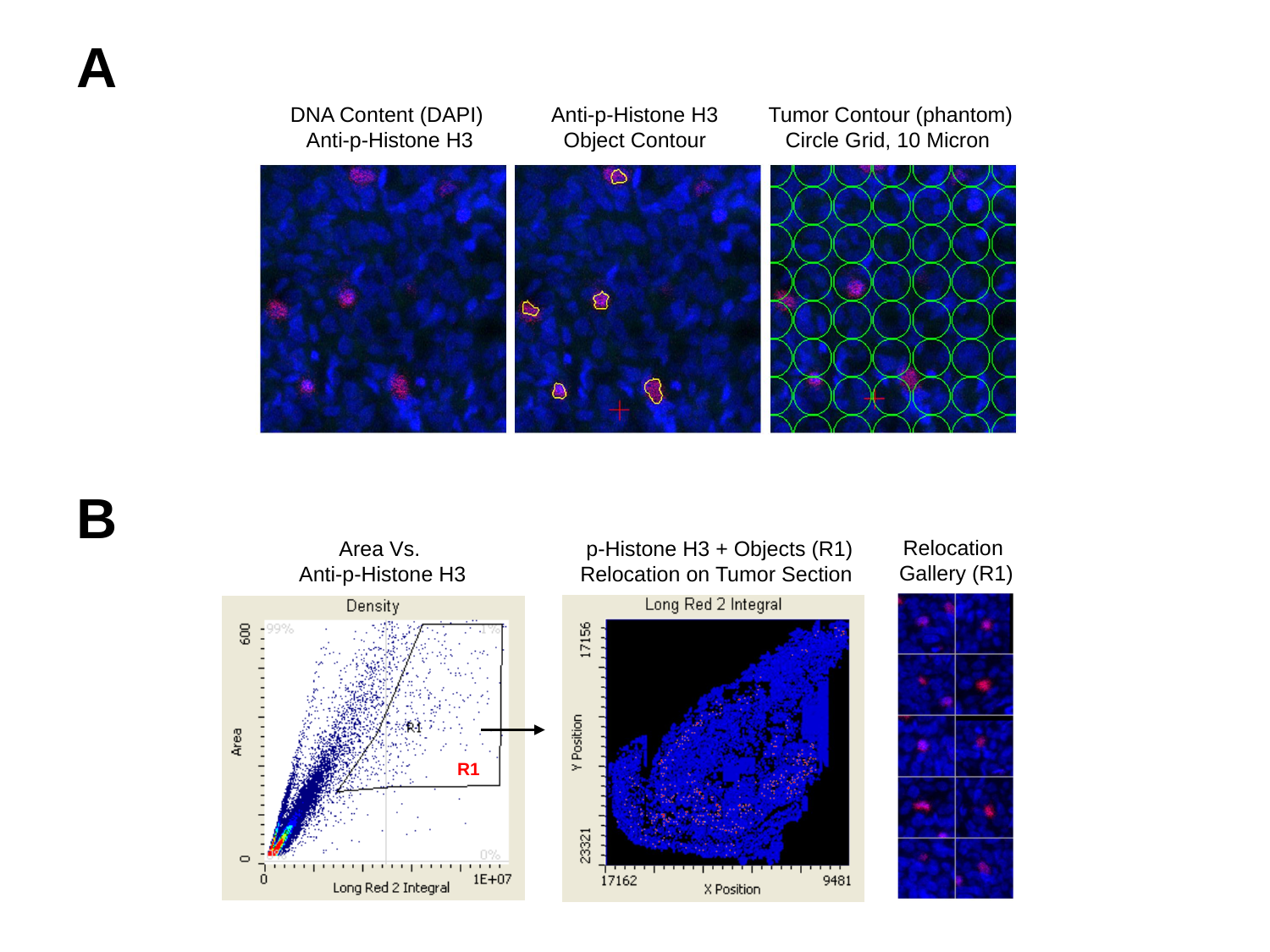

A
DNA Content (DAPI)
Anti-p-Histone H3
Anti-p-Histone H3
Object Contour
Tumor Contour (phantom)
Circle Grid, 10 Micron
Relocation
Gallery (R1)
Area Vs.
Anti-p-Histone H3
p-Histone H3 + Objects (R1)
Relocation on Tumor Section
R1
B
