# Supplementary material for: AMG 900, a potent inhibitor of aurora kinases causes pharmacodynamic changes in p-Histone H3 immunoreactivity in human tumor xenografts and proliferating mouse tissues
Source: J Transl Med. 2014 Nov 4;12:307. doi: 10.1186/s12967-014-0307-x (PMC4221688; doi:10.1186/s12967-014-0307-x)
Supplement: Additional file 3: Figure S3. — LSC segmentation scheme Tumor FNAs. (A) (Upper panel) Cytospun deposited COLO 205 FNAs were immunostained with an anti-p-Histone H3 antibody and counterstained with DAPI. Representative scanned image fields and cellular segmentation based on DAPI (nuclear stain) and positive p-Histone H3 events (red). Lower panel from left show representative cytometric gating plot to exclude aggregates and identify single events, the cell cycle profile (middle plot), and p-Histone H3 (red) in G2M cells (right plot). (B) Representative mock FNA from a human primary breast tumor. Cytospun deposited FNAs were immunostained with an EpCAM antibody, an anti-p-Histone H3 antibody and counterstained with DAPI. Representative scanned image fields and cellular segmentation based on DAPI (nuclear stain). Lower panel from left show representative cytometric gating plot to identify EpCAM positive and negative events, the cell cycle profile (middle plot) for both populations and and p-Histone H3 (red) in G2M cells of EpCAM positive subset cells (right plot). [file 12967_2014_307_MOESM3_ESM.pptx]

## Slide 1
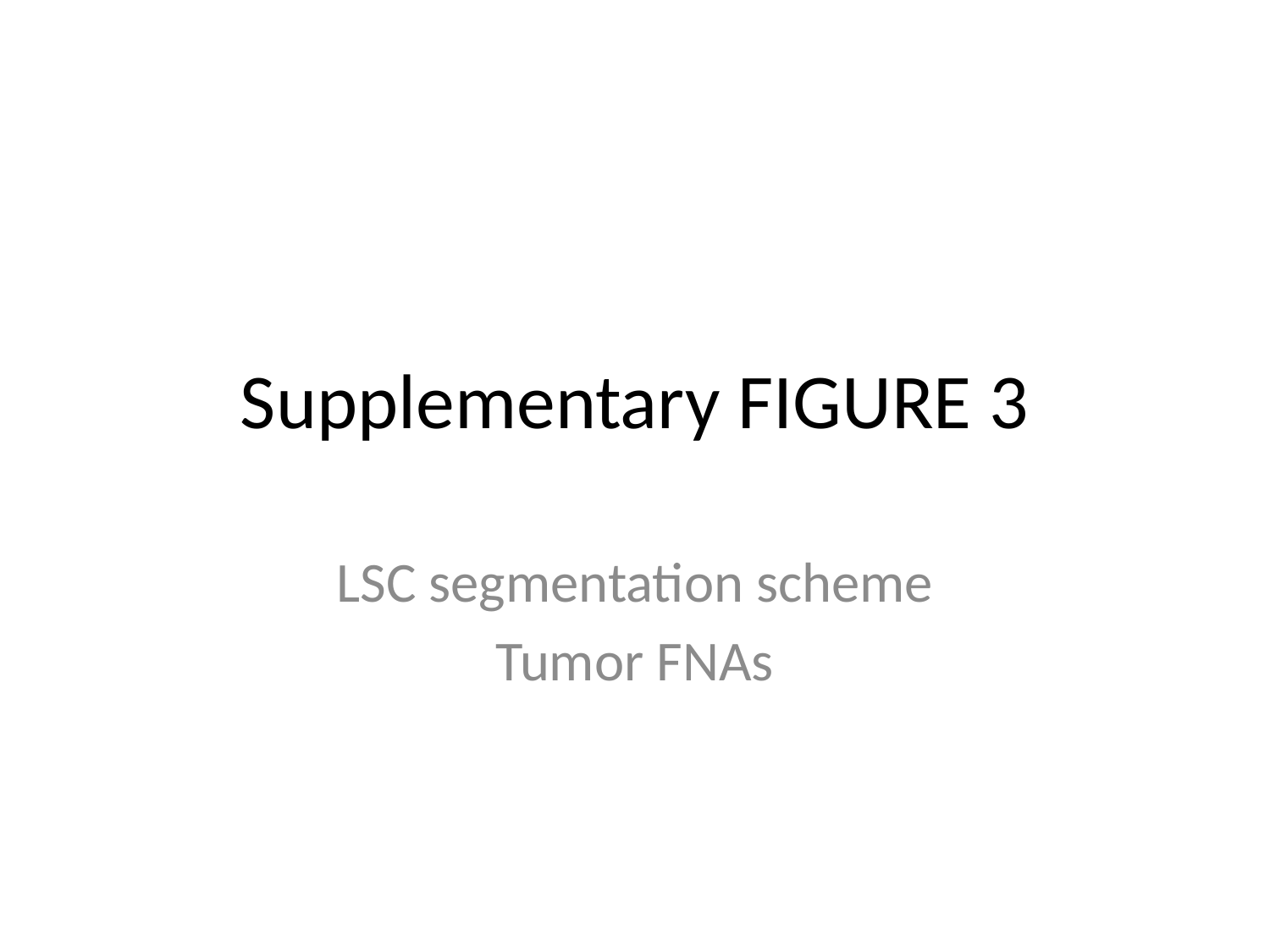

# Supplementary FIGURE 3
LSC segmentation scheme
Tumor FNAs

## Slide 2
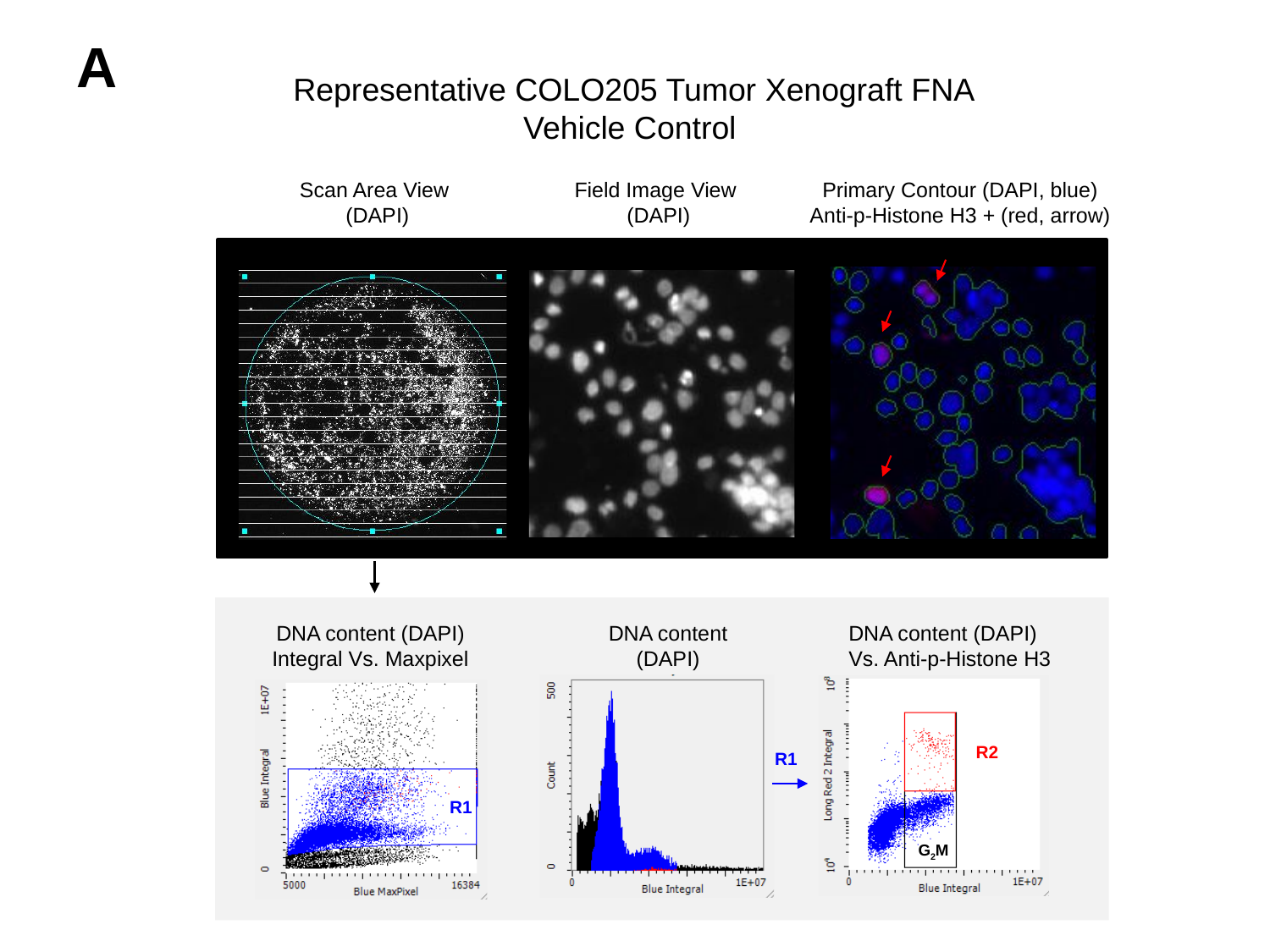

A
Representative COLO205 Tumor Xenograft FNA
Vehicle Control
Scan Area View
(DAPI)
Field Image View
(DAPI)
Primary Contour (DAPI, blue)
Anti-p-Histone H3 + (red, arrow)
DNA content
(DAPI)
DNA content (DAPI)
Integral Vs. Maxpixel
DNA content (DAPI)
Vs. Anti-p-Histone H3
R2
R1
R1
G2M

## Slide 3
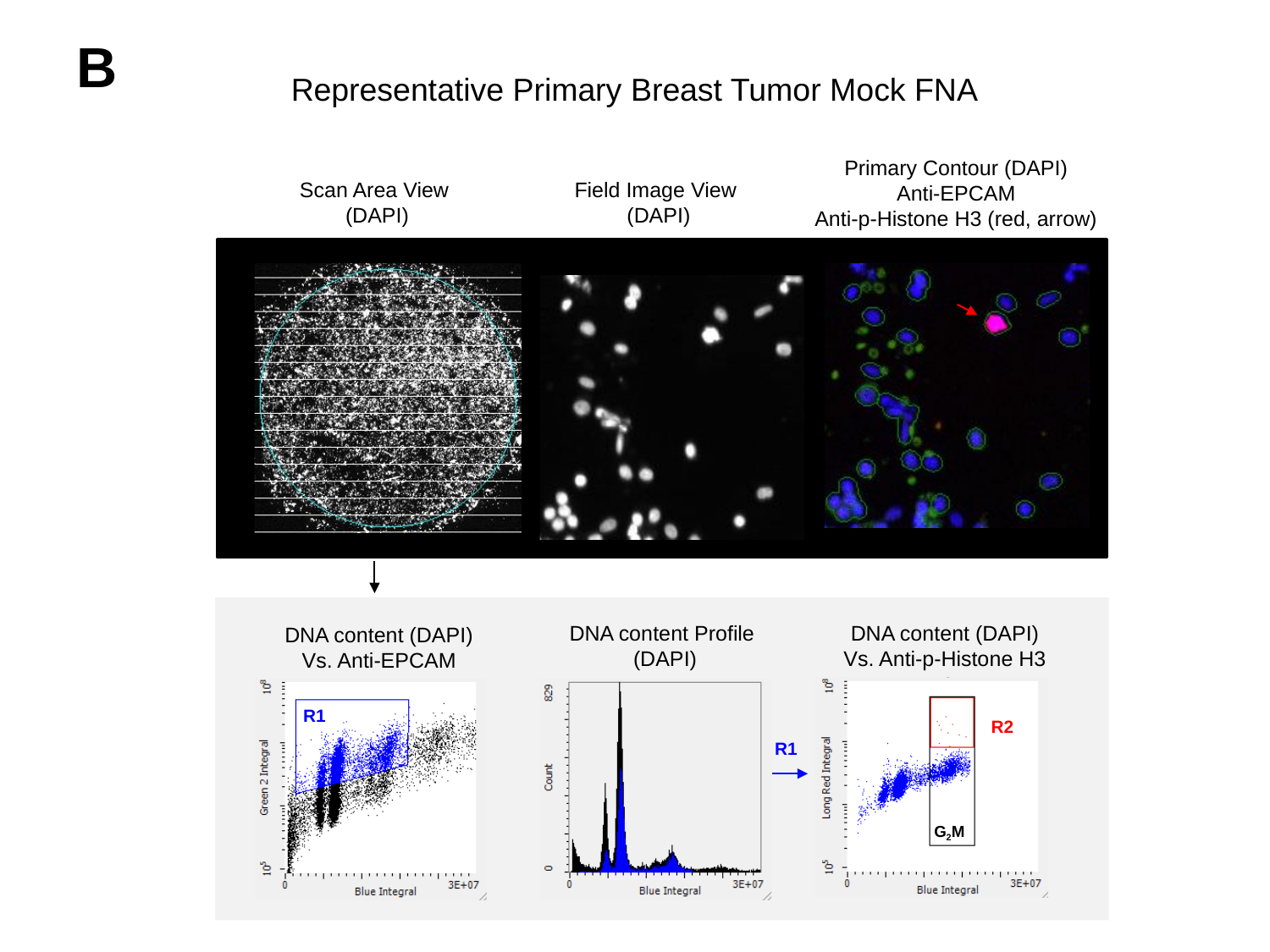

B
Representative Primary Breast Tumor Mock FNA
Primary Contour (DAPI)
Anti-EPCAM
Anti-p-Histone H3 (red, arrow)
Scan Area View
(DAPI)
Field Image View
(DAPI)
DNA content Profile
(DAPI)
DNA content (DAPI)
Vs. Anti-p-Histone H3
DNA content (DAPI)
Vs. Anti-EPCAM
R1
R2
R1
G2M
